# Beiträge zur Pathologie und pathologischen Anatomie des Centralnervensystems

**Published:** 1899-06

**Authors:** 


**Beiträge zur Pathologie und pathologischen Anatomie des Centralnervensystems.** Von Dr. ARNOLD PICK. Pp. viii., 324. Berlin: S. Karger. 1898.

This book is made up of a number of essays on neurological subjects and reports of cases interesting either from the clinical or pathological side, and mostly from both. The cases, which are fully and clearly reported, are important as tending, many of them, to elucidate obscure or unsettled points in our knowledge of the pathology of the nervous system. In each instance there is a full critical commentary of the features of interest, and the references to the literature of the subject are unusually complete. In the first twelve papers, which, comprising about half the volume, deal with various kinds of aphasia, almost entirely sensory aphasia, will be found recorded many important cases, and special mention may be made of the studies of subcortical sensory aphasia, of the significance in localisation of quadrant hemianopsia, and of Wernicke's conduction-aphasia. A series of five papers describe various arrests of formation and malformations of the spinal cord; these are very copiously and well illustrated. Three other chapters are devoted to a case of disease of the interolivary layer, and to the consideration of degenerations of the comma-shaped tract in the posterior columns of the spinal cord, and of von Bechterew's "Olivenbündel." Two cases of tumour of the corpus callosum lead to a discussion on the diagnosis of tumours in this situation, and there are also essays on the return of the knee-jerk in some cases of disease of the posterior columns, and on the clinical features of tabes dorsalis when it occurs in young subjects.

The numerous and well-executed figures of the pathological changes form quite a feature of the book, and add to its value, which largely consists in the number of original observations on cases which have been carefully studied, and which form an important contribution to neurological literature.

